# Improving the baking quality of bread wheat by genomic selection in early generations

**DOI:** 10.1007/s00122-017-2998-x

**Published:** 2017-10-23

**Authors:** Sebastian Michel, Christian Kummer, Martin Gallee, Jakob Hellinger, Christian Ametz, Batuhan Akgöl, Doru Epure, Franziska Löschenberger, Hermann Buerstmayr

**Affiliations:** 1 Department for Agrobiotechnology (IFA-Tulln), Institute for Biotechnology in Plant Production, University of Natural Resources and Life Sciences, Vienna (BOKU), Konrad-Lorenz-Str. 20, 3430 Tulln, Austria; 2 Versuchsanstalt für Getreideverarbeitung, Österreichische Mühlenvereinigung e.V, Prinz-Eugen-Straße 14/1/4, 1040 Vienna, Austria; 3 Saatzucht Donau GesmbH. & CoKG, Saatzuchtstrasse 11, 2301 Probstdorf, Austria; 4 ProGen Seed A.Ş., Büyükdalyan Mah. 2. Küme evler Sok., No: 49, 31001 Antakya, Hatay Turkey; 5 Probstdorfer Saatzucht Romania SRL, Str. Siriului Nr.20, sect. 1, Bucuresti, Romania

## Abstract

**Key message:**

**Genomic selection shows great promise for pre-selecting lines with superior bread baking quality in early generations, 3** **years ahead of labour-intensive, time-consuming, and costly quality analysis.**

**Abstract:**

The genetic improvement of baking quality is one of the grand challenges in wheat breeding as the assessment of the associated traits often involves time-consuming, labour-intensive, and costly testing forcing breeders to postpone sophisticated quality tests to the very last phases of variety development. The prospect of genomic selection for complex traits like grain yield has been shown in numerous studies, and might thus be also an interesting method to select for baking quality traits. Hence, we focused in this study on the accuracy of genomic selection for laborious and expensive to phenotype quality traits as well as its selection response in comparison with phenotypic selection. More than 400 genotyped wheat lines were, therefore, phenotyped for protein content, dough viscoelastic and mixing properties related to baking quality in multi-environment trials 2009–2016. The average prediction accuracy across three independent validation populations was *r* = 0.39 and could be increased to *r* = 0.47 by modelling major QTL as fixed effects as well as employing multi-trait prediction models, which resulted in an acceptable prediction accuracy for all dough rheological traits (*r* = 0.38–0.63). Genomic selection can furthermore be applied 2–3 years earlier than direct phenotypic selection, and the estimated selection response was nearly twice as high in comparison with indirect selection by protein content for baking quality related traits. This considerable advantage of genomic selection could accordingly support breeders in their selection decisions and aid in efficiently combining superior baking quality with grain yield in newly developed wheat varieties.

**Electronic supplementary material:**

The online version of this article (doi:10.1007/s00122-017-2998-x) contains supplementary material, which is available to authorized users.

## Introduction

The genetic improvement of baking quality is one of the grand challenges in winter wheat breeding due to its complex inheritance pattern, which is governed mainly by wheat storage proteins, foremost the prolamins gliadin and glutenin (Payne 1987; Shewry et al. 1995, 2003) as well as their interaction with other fractions like the puroindolins that confer grain hardness (Bekes 2012a; Quayson et al. 2016; Würschum et al. 2016). The quality of these wheat storage proteins can be determined by various measurements, amongst others by dough rheological tests that assess the viscoelastic and mixing properties during dough preparation that are important for food-processing and baking of various products like bread, cookies, and pastry. Baking tests and dough rheology are furthermore part of official variety test and registration in various countries to offer varieties with fitting combinations of quality characteristics. A major obstacle is thereby that the assessment of the associated traits often involve time-consuming, labour-intensive, and costly testing as well as the too low amount of grains that is available per genotype in early breeding generations, thus sophisticated quality tests have usually been postponed to the very last phases of variety development.

Hence, selection decisions for baking quality improvement are beforehand guided by indirect phenotypic selection based on correlated traits like protein content. Protein quantity assessed in this way explains however merely a limited part of the genetic variation observed for traits related to baking quality, for which loci associated with the composition of the wheat storage proteins gliadin and glutenin play a major role (Payne et al. 1987; Lukow et al. 1989; Rogers et al. 1989). A pre-selection of lines in early generations by markers linked to the known *Glu*-*1* and *Glu*-*3* glutenin loci is accordingly an interesting option (Eagles et al. 2002; Zheng et al. 2009; Krystkowiak et al. 2016), but there are few successful reports of such an approach (Kuchel et al. 2007), and the respective markers have to be combined with additional small-scale tests to achieve a reasonable prediction accuracy for selection (Oury et al. 2010). Such a marker-assisted selection focuses mainly on major quantitative trait loci (QTL) that explain a substantial but limited amount of the underlying genetic variance, while most traits of interest in plant breeding show a polygenic inheritance and are, therefore, controlled mostly by many minor QTL.

Genomic selection has been implemented in recent years into many national and international wheat breeding programs (Guzmán et al. 2016a; He et al. 2016; Michel et al. 2016) to additionally target these small effect loci influencing quantitative traits with genome-wide distributed markers in genetically fingerprinted training and selection populations. Training populations in applied wheat breeding programs are normally comprised of advanced breeding material that has been thoroughly phenotyped for grain yield, disease resistances and numerous milling and baking quality traits (Guzmán et al. 2016a; He et al. 2016; Michel et al. 2016). The phenotypic information of the selection population is on the other hand very limited, and genomic estimated breeding values for numerous traits of interest can be derived for these genotypes via their genetic relationship with the training population in order to support breeders in their selection decisions (Heffner et al. 2009; Heslot et al. 2015). This promising selection method could be especially valuable for baking quality related parameters such as dough rheological traits whose assessment requires costly, labour-intensive and time-consuming tests. The aims of this study were thus (1) to investigate the prospect of genomic selection for these laborious to phenotype quality traits, (2) enhancing this approach by integrating prior knowledge about trait correlations and genetic architecture, and (3) compare the selection response of direct and indirect phenotypic with genomic selection.

## Materials and methods

### Plant material and phenotypic data

We analyzed a population of 840 genotyped winter bread wheat lines (*Triticum aestivum* L.), which was derived from multiple families and selected by the pedigree method until the F_4:6_ and F_5:7_ generation or generated by the double haploid method. Different subpopulations of these lines were phenotyped in multi-environment trials at locations in Austria, Hungary, Serbia, Croatia, Romania and Turkey from 2009 to 2016. Grain samples were collected and milled from 401 lines with a Quadrumat Junior milling system according to the method AACC26-50 approved by the American Association of Cereal Chemists (AACC 2000). The resulting flour samples were employed to create a dough rheological profile of each line, starting with the dough-mixing properties that were assessed by a Farinograph (Brabender GmbH and Co KG) equipped with a 300 g mixing bowl. The optimal water uptake of each flour sample was estimated in a preliminary test on a subsample of 100 g flour until it reached a dough consistency of 500 farinogram units (FU) according to the standard procedure AACCI 54e21 (AACC 2000). The dough development time was measured as the time in minutes from the first water uptake until the dough began to soften due to intensive mixing in the main test. Dough stability was assessed as the timeframe between which the kneading curve first intersected and left the 500 FU borderline, and the farino quality number was calculated as the time point when the dough consistency fell 30 FU after reaching its peak. Thereafter, the Extensograph (Brabender GmbH and Co KG) was used to determine the viscoelastic properties of the flour samples according to AACCI 54-10.01 (AACC 2000) of which the extensibility (mm), resistance to extension at 50 mm in extensogram units (EU), and the area under the curve, i.e., the dough energy (cm^2^) after a 135-min resting time were of prior interest in this study. The protein content (%) was determined by near infrared spectroscopy (NIRS; FOSS GmbH) for all 840 lines directly at harvest.

The measurement of baking quality by mixing and viscoelastic tests is typically costly, labour-intensive and time-consuming, thus the obtained phenotypic records from the 401 lines that were subject to dough rheological analysis were highly unbalanced between trials, and the data from different trials were mainly connected by several common check varieties replicated in each of the completely randomized trial designs. The other 439 lines were on the other hand thoroughly tested in multi-environment trials, and orthogonally phenotyped for their protein content across various locations in the above-described target population of environments from 2009 to 2013. Hence, they provided an additional source of information for a rapidly to assess quality parameter that is routinely generated in many wheat breeding programs.

### Statistical analysis of phenotypic data

The phenotypic analysis for the 401 lines with dough rheological profiles was conducted for each trial separately in order to determine the heritability based on:

1 $$h^{2} = {{\sigma_{\text{G}}^{2} } \mathord{\left/ {\vphantom {{\sigma_{\text{G}}^{2} } {\left( {\sigma_{\text{G}}^{2} + \frac{1}{2}{\text{MVD}}} \right)}}} \right. \kern-0pt} {\left( {\sigma_{\text{G}}^{2} + \frac{1}{2}{\text{MVD}}} \right)}},$$

where $\sigma_{\text{G}}^{2}$ designates the genetic variance and ${\text{MVD}}$ the mean variance of a difference of the BLUEs (Piepho and Möhring 2007). Trials with a heritability smaller than 0.1 were excluded from further analysis. This liberal threshold was chosen due to the above-mentioned circumstances. However, in some trials none of the lines were replicated, thus an estimation of the data quality was not possible in these cases. They were nevertheless used with the other trials for a one-step analysis across trials where each trait was analysed separately using a linear mixed model of the form:

2 $$y_{ij} = \mu + g_{i} + t_{j} + e_{ij} ,$$

where $y_{ij}$ are the phenotypic records, $\mu$ is the grand mean, and $g_{i}$ is the effect of the *i*th line. The effect of the *j*th trial $t_{j}$ was fixed and $e_{ij}$ designates the residual effect. The residual variance incorporated both the trial by line interaction variance and the residual effect and could be estimated via the replicated entries within or across trials and was assumed to follow a normal distribution with ${\mathbf{e}} \sim N(0, {\mathbf{I}}\sigma_{\text{e}}^{2} )$. Five different sets of lines with dough rheological profiles were thereby analysed separately: a basis population containing 191 lines that was tested from 2009 to 2013, three independent validation populations tested in the individual years 2014–2016, respectively, and finally a dataset containing all 401 lines tested from 2009 to 2016. The total number of lines in the three independent validation populations was 210, with some lines occurring in several years (Table S1 Online Resource 1).

The additional 439 lines tested in multi-environment trials from 2009 to 2013, were analysed for their performance with regard to the protein content following a two stage analysis strategy. Each individual yield trial was first analysed with various models correcting for row and/or column effects as well as with an autoregressive variance–covariance structure (Burgueño et al. 2000). The best model was chosen by Akaike’s Information Criterion (AIC) to calculate best linear unbiased estimates (BLUE), while trials with a heritability larger than 0.3 were used for an across trial analysis for each individual year 2009–2013 following formula (2). All phenotypic analyses were conducted using the statistical package ASReml 3 for the R programming environment (R Development Core Team 2016).

### Genotypic data

DNA was extracted following the protocol by Saghai-Maroof et al. (1984) using leaf samples that were collected from F_4:5_ or doubled haploid lines by sampling minimum ten plants per line during early summer. All 840 lines were genotyped using the DarT genotyping-by-sequencing (GBS) approach. Quality control was applied by filtering out markers with a call rate lower than 90%, a minor allele frequency smaller than 0.05, and more than 10% of missing data. An MVN-EM algorithm (Poland et al. 2012) was employed to impute missing data of the remaining 7687 markers, and their pair-wise correlation was used as an ad hoc measure of linkage disequilibrium. One marker from each marker pair that exceeded the *r* ^2^ = 0.8 threshold was dropped at random in order to remove strongly correlated predictor variables that would not contribute further to the prediction accuracy but elongate computation time, which resulted in a final set of 4598 markers.

A map position was available for 2637 of these markers with an average coverage of one marker every 1.8 cM. The usage of phenotypic data from the genotyped lines in a higher generation than the F_4:5_ was expected to introduce a small error due to a minor change in average heterozygosity, which was nevertheless seen to be acceptable considering the cost–benefit ratio of re-genotyping all lines in the advanced generations. Additionally, a subset of 444 lines was screened for their allelic state at the high-molecular weight glutenin subunit loci *Glu*-*A1*, *Glu*-*B1*, and *Glu*-*D1* by sodium dodecyl sulphate polyacrylamide gel electrophoresis (SDS-PAGE). The *missForest* algorithm (Stekhoven and Bühlmann 2012) was used to impute the missing values of the other lines in a chromosome-wise manner, employing both the GBS and SDS-PAGE markers.

### Single-trait genomic and marker-assisted selection

**Fig. 1:**
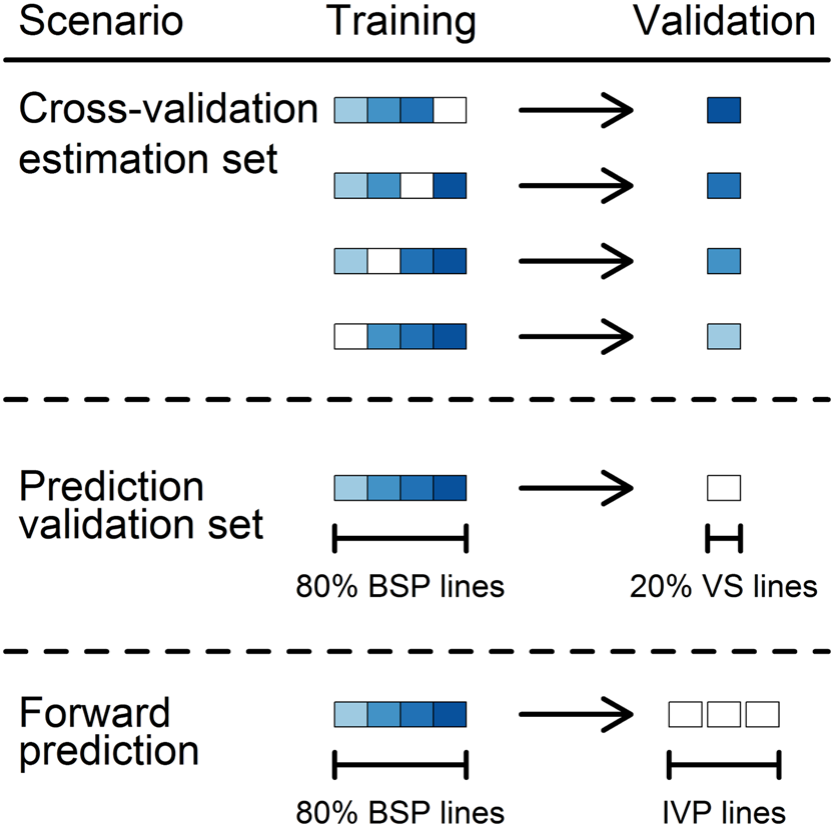
Prediction scenarios used for the fourfold cross-validation within estimation set used for the GWAS in the basis population 2009–2013 (BSP), prediction of the validation set of the 20% of lines left-out for the GWAS (VS), and the forward prediction of the three independent validation populations 2014–2016 (IVP)

First we investigated the merit of predicting each single trait separately with marker effect estimations based on a ridge regression best linear unbiased prediction (RR-BLUP) model both for genomic and marker-assisted selection:

3 $${\mathbf{y}} = {\mathbf{Xb}} + {\mathbf{Zu}} + {\mathbf{e}},$$

where ${\mathbf{y}}$ is an Nx1 vector of BLUEs obtained in the phenotypic analysis, ${\mathbf{b}}$ is a vector of *F* fixed effects and ${\mathbf{X}}$ its corresponding *N* × *F* design matrix. ${\mathbf{Z}}$ is a *N* × *M* matrix, which coded the *M* markers as either + 1 or − 1 for homozygous loci and 0 for heterozygous loci. Random marker effects were assumed to follow a normal distribution ${\mathbf{u}}\sim N(0, {\mathbf{I}}\sigma_{\text{u}}^{2} )$ with variance $\sigma_{\text{u}}^{2}$ and ${\mathbf{e}}\sim N(0, {\mathbf{I}}\sigma_{\text{e}}^{2} )$. The basis population with the 191 lines tested from 2009 to 2013 was used to compare different selection strategies by 100 times sampling 80% of the lines into an estimation set and using the left-over 20% as validation set (Fig. 1).

Three options were considered for exploiting prior knowledge about the genetic architecture of the dough rheological parameters by a marker-assisted selection strategy:

1. Prediction using the markers linked with the *Glu*-*1* loci on chromosomes 1A, 1B, and 1D.
2. Performing a genome-wide associations study (GWAS) with the K model and previously determined population parameters (Yu et al. 2006; Zhang et al. 2010) in the estimation set with markers of known map position and subsequent prediction with the three most significant markers.
3. Combining the known *Glu*-*1* loci markers with the de novo identified markers by GWAS.

The genome-wide distributed GBS markers opened together with the de novo found markers and prior knowledge of the *Glu*-*1* loci another two interesting options for a genomic selection approach:

1. Fitting the RR-BLUP model with all 4598 markers as random effects for predicting the various dough rheological traits.
2. Including the three marker sets used for marker-assisted selection as fixed effects into the RR-BLUP model, thereby excluding them from the random effects matrix.

The latter method has sometimes been termed weighted genomic best linear unbiased prediction (W-BLUP) and has shown a superior performance when major QTL are known (Bernardo 2014; Zhao et al. 2014; Arruda et al. 2016; Spindel et al. 2016). The prediction accuracy for both genomic and marker-assisted selection was calculated as the correlation between the genomic estimated breeding values and the phenotypic observations, i.e., BLUEs, while using the estimation set as training population for different prediction model validation strategies (Fig. 1):

1. Prediction by fourfold cross-validation within the estimation set used for the GWAS.
2. Prediction of the validation set, i.e., the left-out 20% of lines.
3. Forward prediction of lines tested in 2014–2016.

The forward prediction was thereby done for three independent validation populations that consisted of 70, 79, and 125 lines for which a dough rheological profile was generated in 2014, 2015, and 2016, respectively. It should be stressed out at this point that in the first scenario of cross-validation within the estimation set used for the GWAS, predictors are selected before leaving observations out. The left-out observations are however supposed to be unobserved for a correct application of cross-validation, and marker selection has to be conducted after leaving such validation data out (Hastie et al. 2009). Nevertheless, this wrong statistical method has been used in several recent publications about genomic selection in plant breeding. Hence, we like to highlight the outcome of such a procedure in comparison with correctly applied cross-validation and independent validation in this study, especially as the potential consequences could be dramatically when prediction models are actually employed for conducting selection decisions in applied plant breeding programs. All models for the single-trait genomic and marker-assisted selection were fitted with the package rrBLUP (Endelman 2011) as implemented in the R programming environment (R Development Core Team 2016).

### Multi-trait genomic selection

The protein content can rapidly be determined for a large number of lines, and is thus routinely assessed in many wheat breeding programs. The large amount of available phenotypic data on protein content and the correlation with other quality traits made it an interesting variable for examining different bivariate models including both protein content and dough rheological parameters. The benefit of this approach was investigated with a cross-validation approach using the entire population of 401 dough rheological analysed lines from 2009 to 2016. One-third of the lines were randomly sampled into a validation population, one-third comprised the training population, and the last third formed a population of additional lines, i.e., an additional source of information for each of the 100 cross-validation replicates. A genomic best linear unbiased prediction model (G-BLUP) was fitted for each single-trait to determine the baseline prediction accuracy:

4 $${\mathbf{y}} = {\mathbf{Xb}} + {\mathbf{Zg}} + {\mathbf{e}},$$

where ${\mathbf{y}}$ is an *N* × 1 vector of BLUEs obtained in the phenotypic analysis, ${\mathbf{g}}$ is an *N* × 1 vector of line effects with the genetic variance $\sigma_{\text{G}}^{2}$ and ${\mathbf{g}} \sim N(0, {\mathbf{K}}\sigma_{\text{G}}^{2} )$ as well as the random effect design matrix ${\mathbf{Z}}$. The fixed effect matrix ${\mathbf{X}}$ and the corresponding vector ${\mathbf{b}}$ modeled merely the grand mean in this single-trait prediction case. Additionally, the shrinkage parameter given by $\lambda^{2} = \sigma_{\text{e}}^{2} /\sigma_{\text{g}}^{2}$ with the residual variance $\sigma_{\text{e}}^{2}$ that followed ${\mathbf{e}} \sim N(0, {\mathbf{I}}\sigma_{\text{e}}^{2} )$ was recorded for each trait and cross-validation replicate. The necessary genomic relationship matrix ${\mathbf{K}}$ was computed according to Endelman and Jannink (2012):

5 $${\mathbf{K}} = {\mathbf{WW}}^{\text{T}} /2\varSigma (p_{k} - 1)p_{k} ,$$

where ${\mathbf{W}}$ is a centered *N* × *M* marker matrix of the *i* lines with $W_{ik} = Z_{ik} + 1 - 2p_{k}$ and $p_{k}$ being the allele frequency at the *k*th locus. The genomic relationship matrix ${\mathbf{K}}$ was utilized again for fitting a multi-trait model which always contained the protein content as correlated trait and the varying rheological traits of interest:

6 $${\mathbf{y}}_{\varvec{t}} = {\mathbf{X}}_{\varvec{t}} {\mathbf{b}}_{\varvec{t}} + {\mathbf{M}}_{\varvec{t}} {\mathbf{g}}_{\varvec{t}} + {\mathbf{e}}_{\varvec{t}} ,$$

where ${\mathbf{y}}$ is an *N* × *t* vector of BLUEs for *t* traits obtained in the phenotypic analysis, ${\mathbf{g}}_{\varvec{t}}$ is the vector of *N* × *t* line effects with the corresponding random effect design matrix ${\mathbf{M}}_{\varvec{t}}$ and ${\mathbf{g}}_{\varvec{t}} \sim {\text{MVN}}\left( {0, \mathop \varSigma \nolimits_{\text{g}} \otimes {\mathbf{K}}} \right)$ with the completely unstructured variance–covariance matrix $\mathop \varSigma \nolimits_{\text{g}}$ of the form:

7 $$\left( {\begin{array}{*{20}c} {\sigma_{{{\text{g}}_{1} }}^{2} } & {\sigma_{{{\text{g}}_{12} }} } \\ {\sigma_{{{\text{g}}_{12} }} } & {\sigma_{{{\text{g}}_{2} }}^{2} } \\ \end{array} } \right),$$

where $\sigma_{{{\text{g}}_{1} }}^{2}$ and $\sigma_{{{\text{g}}_{2} }}^{2}$ are the genetic variance of the first and second trait, respectively, and $\sigma_{{{\text{g}}_{12} }}$ is the genetic covariance between both traits. The variance of the residual effect followed ${\mathbf{e}}_{\varvec{t}} \sim {\text{MVN}}\left( {0, \mathop \varSigma \nolimits_{\text{e}} \otimes {\mathbf{I}}_{\varvec{N}} } \right)$ where ${\mathbf{I}}_{\varvec{N}}$ is an identity matrix of dimension *N* × *N* and $\mathop \sum \nolimits_{\text{e}}$ the completely unstructured variance–covariance matrix for the residual effect analogues to (7) though with residual variances and covariance between traits. The fixed effect part ${\mathbf{X}}_{\varvec{t}} {\mathbf{b}}_{\varvec{t}}$ of model includes now a fixed effect ${\mathbf{b}}_{\varvec{t}}$ with two levels for the respective traits.

However, multi-trait models suffer often from a high computational demand, very long computational times, and convergence problems that might increase when predictions are done with larger training population sizes than the ones in this study. Hence, we studied the possibility to exploit the correlation between the protein content and rheological parameters by combining their single-trait predictions by a selection index. For this purpose, genomic estimated breeding values were computed for the training and validation populations by model (4) for the protein content in a first step. The vector of derived genomic estimated breeding values of the protein content was subsequently included as a fixed effect into model (4) when predicting the dough rheological traits. The final genomic estimated breeding values of each individual line for the respective dough rheological trait was computed by:

8 $${\text{GEBV}}_{i} = x_{i} b_{\text{Protein}} + g_{i} ,$$

with $g_{i}$ being the random genetic effect of the *i*th line, $x_{i}$ being the genomic estimated breeding value for protein content of the *i*th line, and $b_{\text{Protein}}$ the estimated fixed effect of the protein content. The suggested method exploits the principle that the highly heritable and well predicted protein content (Michel et al. 2016) is associated with some of the involved rheological parameters (Zanetti et al. 2001; Bordes et al. 2008; Tsilo et al. 2013) in a given training population, while the correlation for other parameters will be less pronounced and thus the index weight as regulated by $b_{\text{Protein}}$ will accordingly lose in importance in these cases.

The two presented multi-trait methods were compared among each other with the single-trait G-BLUP model in four scenarios, each reflecting a situation that might arise in a wheat breeding program:

1. Phenotypic data of dough rheological and correlated traits, i.e., protein content is only available for the training population.
2. The validation/selection population has already been phenotyped for protein content.
3. A large number of additional lines has been genotyped and phenotyped for protein content, though were not advanced for further testing. Nevertheless, these lines are an integral part of training populations in breeding programs with genomic selection, and have the ability to double the training population size of a correlated trait such as protein content in multi-trait prediction models.
4. Phenotypic data of dough rheology is merely available for the training population, but protein content is phenotyped for a large number of additional lines as well as the validation and training population.

The single-trait and multi-trait G-BLUP models were fitted with the package sommer (Covarrubias-Pazaran 2016) for R (R Development Core Team 2016).

### Forward prediction and response to selection

All 191 lines of the basis population tested from 2009 to 2013 were finally utilized to build a training population for a forward prediction of the three independent validation populations applying all previously described models as well as the combination of W-BLUP with multi-trait models for predicting the line performance in each individual validation population tested in 2014, 2015, and 2016, respectively. The explained genetic variance of each candidate marker to be integrated into the W-BLUP model was estimated by a fivefold cross-validation with 100 replicates within the basis population similar to Würschum and Kraft (2013). The employed procedure was very similar to the previously described method for the single-trait predictions using a marker-assisted selection approach. Briefly, a linear model was fitted with an estimation set of lines, excluding lines in one of the folds as validation set at a time whose performance was predicted by:

9 $${\hat{\mathbf{y}}}_{\text{TS}} = {\mathbf{X}}_{\text{TS}} {\hat{\mathbf{b}}}_{\text{ES}} ,$$

where ${\mathbf{X}}_{\text{TS}}$ is the vector of marker information from the test set and ${\hat{\mathbf{b}}}_{\text{ES}}$ is the vector of genetic effects derived from the estimation set. The explained genetic variance of each marker was subsequently calculated as the adjusted squared correlation coefficient $\varvec{R}_{\text{adj}}^{2}$ of the predicted and observed performance in the validation set divided by the heritability of the investigated trait. The above-described marker-assisted selection strategies were furthermore studied by setting thresholds of the explained genetic variance between 0 and 25% in increments of 0.5% for the inclusion of markers into the W-BLUP model.

The putative response to selection to marker-assisted and genomic selection was furthermore compared with the response to indirect selection by protein content when applying different selection intensities. Selection response was assessed as the relative superiority in average trait performance when selecting a population comprised of the predicted best 10–50% of lines in contrast to the average trait performance of all lines in a given independent validation population. For this purpose, the average performance of the selected population for each individual dough rheological trait was estimated by:

10 $$\hat{\mu }_{{{\text{Sel}}_{i} }} = \mu_{i} + h^{2}_{i} (\mu_{{{\text{Sel}}_{i} }} - \mu_{i} ),$$

where $\mu_{i}$ is the average trait performance of an entire independent validation population, $\mu_{{{\text{Sel}}_{i} }}$ is the average trait performance of the selected lines and $h^{2}_{i}$ is the heritability of the *i*th dough rheological trait. The heritability was set to $h^{2}_{i}$ = 1 for marker-assisted and genomic selection, while it was the respective trait heritability computed by Eq. (1) for indirect selection by the protein content and direct phenotypic selection. The latter was used to predict the response of direct and indirect phenotypic selection across years in order to enable a comparison with the marker-based methods, notwithstanding that too few lines were retested in several years to conduct an empirical assessment. The relative superiority in trait performance of the selected population was subsequently calculated by:

11 $$\hat{\rho }_{{{\text{Rel}}_{i} }} = (\hat{\mu }_{{{\text{Sel}}_{i} }} - \mu_{i} )/\mu_{i} .$$

This estimate was averaged over all independent validation populations and dough rheological traits and compared with the proportion of correctly selected lines by genomic and random selection for every selection intensity investigated in this study. Finally, an example dataset and R Code illustrating the implementation of single and multi-trait models for genomic selection with the R package sommer (Covarrubias-Pazaran 2016) was made available for the interested reader (Online Resource 2, Online Resource 3).

## Results

### Quantitative-genetic parameters and trait correlations

We observed a large range of values for all dough rheological parameters and the protein content (Table 1), thus the quality of lines would stretch across all classes of fodder, baking and elite wheat seen, e.g., in German official trials (Laidig et al. 2016). A high ranking in the baking quality class is usually desirable for bread wheat, and in Austria such quality wheat varieties show generally a development time larger than 4.5 min and stability higher than 6 min with regard to their dough-mixing properties as assessed by the Farinograph (AGES 2016). The studied population showed an average performance similar to the former trait value while it surpassed the requirements for the latter dough rheological parameter, yet it contained lines with both highly desirable and non-desirable dough-mixing properties. Accordingly, the viscoelastic dough properties showed a similar high variation, whereas lines with high values for the dough energy are of special interest as they often demonstrate a favourable performance in baking tests such as high loaf volumes. A large part of this phenotypic variation could be explained by genetic differences, but there remained also a substantial non-genetic part caused either by genotype by environment interaction as well as local variation within the trials. Despite the strong unbalancedness of the trial series with few lines being common across trials, and a simple completely randomized trial design, medium to high heritabilities were achieved for all studied traits (Table 1). Hence, the data quality was sufficient and the lines represented a broad sample of protein quantity and quality expected from wheat breeding programs in early generations where genomic selection would be an interesting method.

**Table 1:** Variance components, heritability, mean and range of the dough rheological parameters and the protein content for the entire population of dough rheologically analyzed lines from 2009 to 2016

Method	Parameter	Summary statistics			Variance components		*h* ^2^
		Min	Mean	Max	$\sigma_{\text{G}}^{2}$	$\sigma_{\text{e}}^{2}$	
Farinograph	Water uptake (%)	52.29	59.16	64.65	2.76	2.27	0.61
	Development (min)	0.97	4.10	11.19	1.26	2.44	0.40
	Stability (min)	0.18	14.26	31.15	25.81	38.92	0.47
	Quality number (× 10 min)	7.08	137.86	351.28	2634.16	4010.98	0.46
Extensograph	Resistance (EU)	132.10	393.00	711.70	3001.35	5565.03	0.40
	Extensibility (mm)	121.70	172.00	220.30	140.56	177.74	0.51
	Energy (cm^2^)	45.12	116.81	192.50	515.45	337.05	0.66
NIRS^a^	Protein content (%)	10.79	13.16	15.53	0.41	0.58	0.48

Genotypic variance $\sigma_{\text{G}}^{2}$, error variance $\sigma_{\text{e}}^{2}$, and heritability *h* ^2^ from the across trial analysis of the protein content and the respective dough rheological parameters. The trial residual and genotype by environment interaction variance are both confounded in the error variance

^a^Near infrared spectroscopy

The relationship between traits might further influence the selection decisions as well as the selection methodological choice in different stages of a breeding program. The genetic and phenotypic correlation was estimated with the same multi-trait model used for genomic prediction, whereby all phenotypic records were available and merely two traits were considered at a time due to the high computational demand. The dough energy was thereby strongly correlated with both the extensibility and resistance to extension although the phenotypic and genetic relationship with the latter trait was much more pronounced (Table 2). Additionally, extensibility and resistance to extension were negatively correlated among themselves and thus a selection based on dough energy would be more promising for a simultaneous improvement of these viscoelastic traits. A similar relationship was found for the dough-mixing properties, where the farino quality number was stronger correlated with both the dough development and stability than both traits among each other, and was accordingly a suitable trait for the combined selection and improvement of these two dough rheological parameters. Dough stability, farino quality number and dough energy were furthermore strongly related to each other, which suggested that partly similar dough rheological properties are assessed by these parameters. Traits like the water uptake and extensibility were on the other hand not as strongly connected to the other above-mentioned traits and built a rather separate correlation network on their own. Apart from the protein quality the protein quantity had also a major influence as indicated by the significant positive phenotypic as well as genetic correlations of protein content with most of the dough rheological traits, which already suggested some merit of including the highly heritable protein content into the prediction models. Notwithstanding, it also shows that dough rheological properties are not solely governed by the protein content and that additional factors such as the high molecular weight glutenin subunit composition of wheat storage proteins influence the baking quality parameters of bread wheat.

**Table 2:** Phenotypic (upper triangle) and genetic correlation (lower triangle) as estimated by a multi-trait G-BLUP model between dough rheological parameters and the protein content in the entire population of 401 dough rheological analyzed lines from 2009 to 2016

	PROT	WAT	DEV	STAB	FQN	EXT	RES	ENG
PROT		0.39	0.45	0.22	0.26	0.35	0.02	0.25
WAT	0.29		0.24	0.00	0.09	− 0.01	− 0.01	− 0.05
DEV	0.85	0.20		0.18	0.24	0.29	− 0.02	0.20
STAB	0.76	0.22	0.78		0.97	0.18	0.33	0.51
FQN	0.86	0.36	0.97	0.99		0.18	0.21	0.37
EXT	0.43	− 0.04	0.66	0.54	0.58		− 0.39	0.32
RES	0.20	0.15	0.15	0.55	0.56	− 0.20		0.57
ENG	0.52	0.02	0.55	0.91	0.91	0.46	0.71	

Correlations are shown for the protein content (PROT), water uptake (WAT), dough development (DEV), dough stability (STAB), farino quality number (FQN), extensibility (EXT), resistance to extension (RES), and dough energy (ENG)

### Genomic selection with candidate loci and de novo found marker-trait associations

**Fig. 2:**
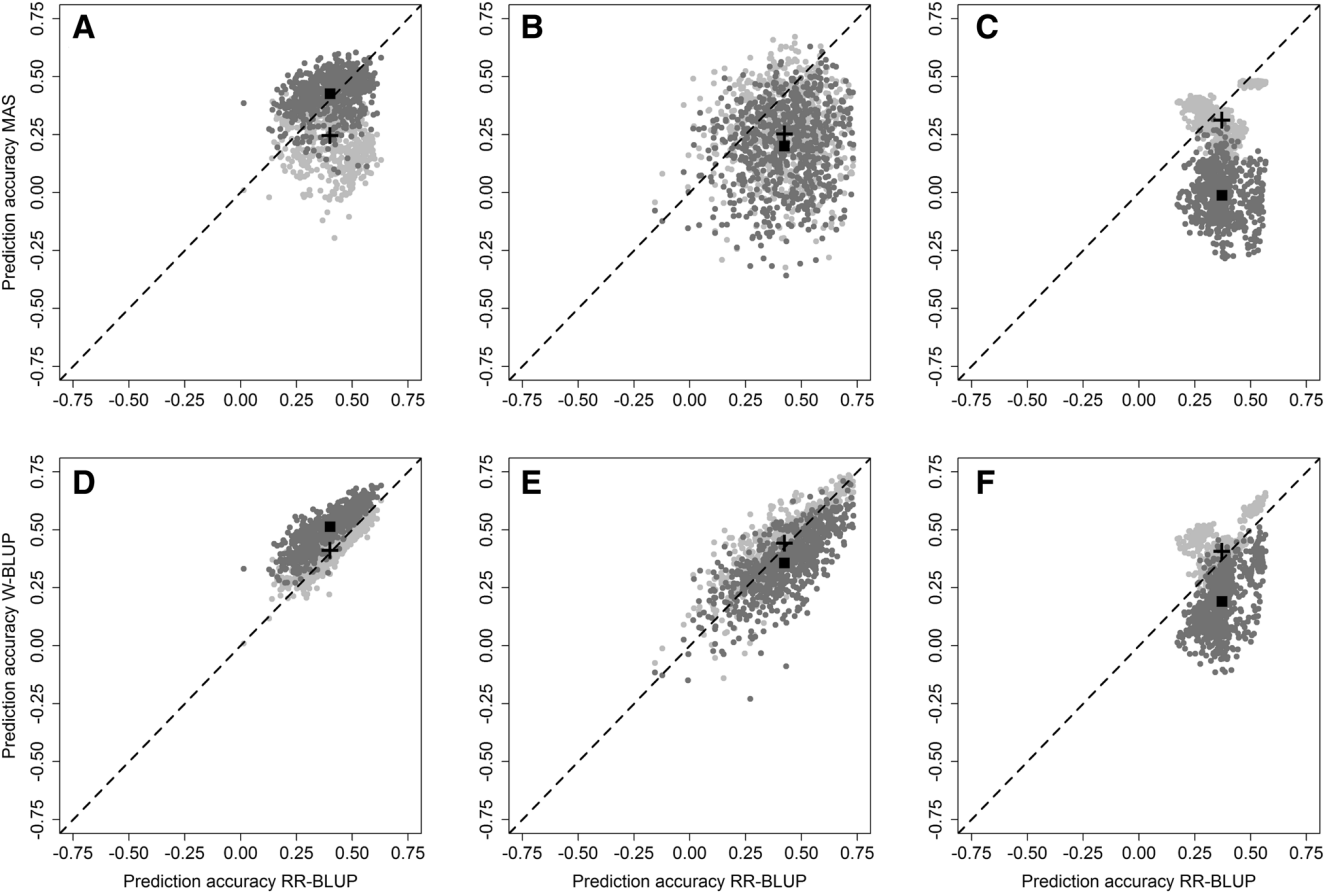
Comparison between marker-assisted (top row) and genomic selection (bottom row) approaches employing markers linked with the *Glu*-*1* loci (pale points) and de novo identified marker-trait associations (dark points), where each point represents a training by validation population by trait combination. Results are shown for the fourfold cross-validation within estimation set used for the GWAS (**a**, **d**), prediction of the validation set of the 20% of lines left-out for the GWAS (**b**, **e**), and the forward prediction of the three independent validation populations 2014–2016 (**c**, **f**). The average across all combinations for methods including the *Glu*-*1* loci (cross) and the de novo identified marker-trait associations (square) is also displayed

The importance of the high molecular weight glutenin subunit composition was emphasized by the results of the fourfold cross-validation within the estimation set used for the GWAS, where the usage of the three markers associated with the *Glu*-*1* loci showed some promise for a marker-assisted selection (Fig. 2a). Nevertheless, the average prediction accuracy for marker-assisted selection with the *Glu*-*1* loci (*r* = 0.25) was still lower than the baseline prediction accuracy of a genomic selection approach (*r* = 0.40), even when including all three *Glu*-*1* loci markers into the prediction model. The employment of de novo found markers by GWAS generally resulted in an higher average prediction accuracy for marker-assisted selection (*r* = 0.43) even surpassing the prediction accuracy of a RR-BLUP model. The combination of both *Glu*-*1* loci and de novo found markers was a slightly more advantageous method (*r* = 0.44), where the latter set of markers most likely modelled genetic relationships within the training population rather than actual marker-trait associations across the training and validation population. This hypothesis was supported by a decrease in prediction accuracy when predicting the validation set of the 20% of lines that were left out for the GWAS (Fig. 2b), where the average prediction accuracy of a marker-assisted selection with the de novo found markers dropped to *r* = 0.21 and the combination was still slightly higher (*r* = 0.26) than employing the *Glu*-*1* loci alone (*r* = 0.25). The dramatic drop in prediction accuracy with the de novo found markers suggested that the models were strongly overfitted when using the same set for estimation and cross-validation in which these marker-trait associations were discovered. The effect was accordingly even more pronounced in the forward prediction of lines tested in 2014–2016 (Fig. 2c): The average prediction accuracy a marker-assisted selection with the *Glu*-*1* loci stayed rather stable (*r* = 0.31), but the predictive ability of models using the de novo found markers completely disappeared (*r* = − 0.02) in this case.

Integrating these de novo found markers as fixed effects into a RR-BLUP model for genomic selection followed the same pattern (Fig. 2d–f), and in the independent validation the prediction accuracy of the according W-BLUP models was approximately halved (*r* = 0.18) in comparison to the baseline RR-BLUP model (*r* = 0.37). Nevertheless the cross-validation and independent validation revealed some merit of including the known *Glu*-*1* loci markers as fixed effects into the RR-BLUP model for constructing a W-BLUP model, as the prediction accuracy could be slightly increased even for the naïve approach when using all *Glu*-*1* loci markers irrespectively of the explained genetic variance for a dough rheological trait. Independent validation resulted, e.g., in an average prediction accuracy across all dough rheological traits of *r* = 0.41 that was slightly higher than the estimated baseline prediction accuracy of *r* = 0.37 obtained with a standard RR-BLUP model. The increase in prediction accuracy by the W-BLUP model was furthermore independent of the population structure that was investigated via principal component analysis (Fig. S1 Online Resource 4).

**Fig. 3:**
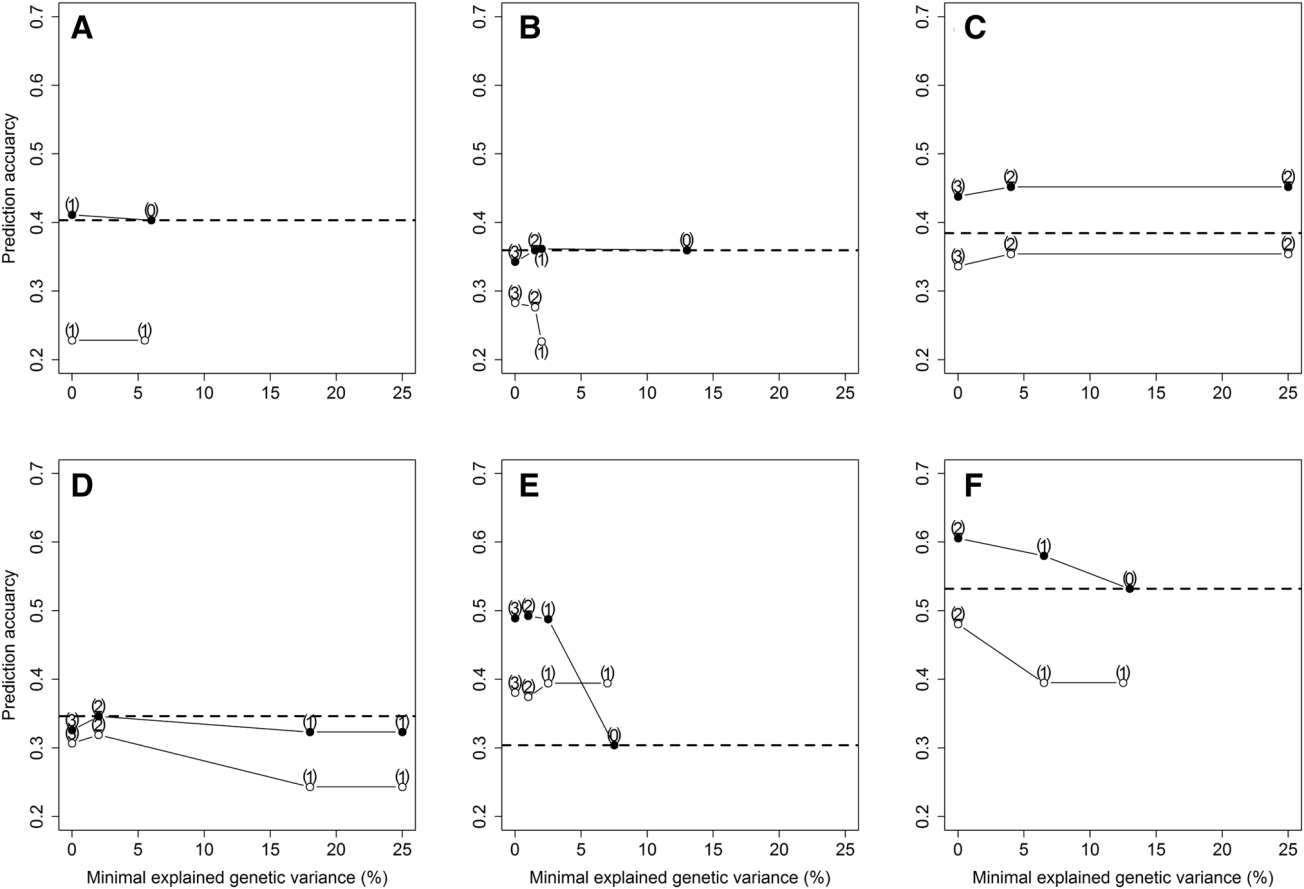
The average prediction accuracy across all three independent validation populations 2014–2016 when training models for marker-assisted selection (open symbols) and integrating the *Glu*-*1* loci as fixed effects into models for genomic selection (closed symbols). Different threshold values based on the explained genetic variance were applied for the water uptake (**a**), dough development (**b**), dough stability (**c**), farino quality number (**d**), resistance to extension (**e**), and the dough energy (**f**). The number of employed markers is highlighted in brackets and the dashed horizontal line represents the baseline prediction accuracy of the RR-BLUP model without including any markers as fixed effects

The fine-tuning of such an approach depended on the threshold value for including the *Glu*-*1* loci into the prediction model when using the entire basis population of 191 lines for training the models (Fig. 3). This advantage was furthermore trait-specific as hardly any benefit was obtained for water uptake, dough development, and quality number but on the other hand a substantial benefit was seen for dough stability, energy, and resistance to extension. The *Glu*-*1* loci did not explain a noteworthy proportion of the genetic variance for dough extensibility, which was expected as this trait is mostly governed by the gliadin protein fraction and was thus not considered for prediction by a W-BLUP model in this study.

The general advantage of a W-BLUP model largely disappeared at thresholds higher than 10% as few of the three assayed *Glu*-*1* loci markers explained such a huge amount of genetic variance of the dough rheological traits. The threshold values were however based on a cross-validation within the basis population 2009–2013 and thus stricter than utilizing the often biased, i.e., over-optimistic values derived from linear models without such a scheme. Finally, we set a threshold of 5.00% explained genetic variance for including the *Glu*-*1* loci markers as a compromise between explained genetic variance and marker number, as, e.g., a clear advantage was already seen by modelling *Glu*-*D1* as fixed effect for predicting resistance to extension in the validation set of the 20% of lines that were left out for the GWAS. These markers would otherwise been excluded by using a stricter threshold of 10.00% as suggested by Bernardo (2014). The average prediction accuracy of the forward prediction was increased by 13% from *r* = 0.39 to *r* = 0.44 when integrating this prior knowledge about the genetic architecture of wheat storage protein composition into the genomic prediction models.

### Multi-trait prediction models and response to selection for baking quality

**Fig. 4:**
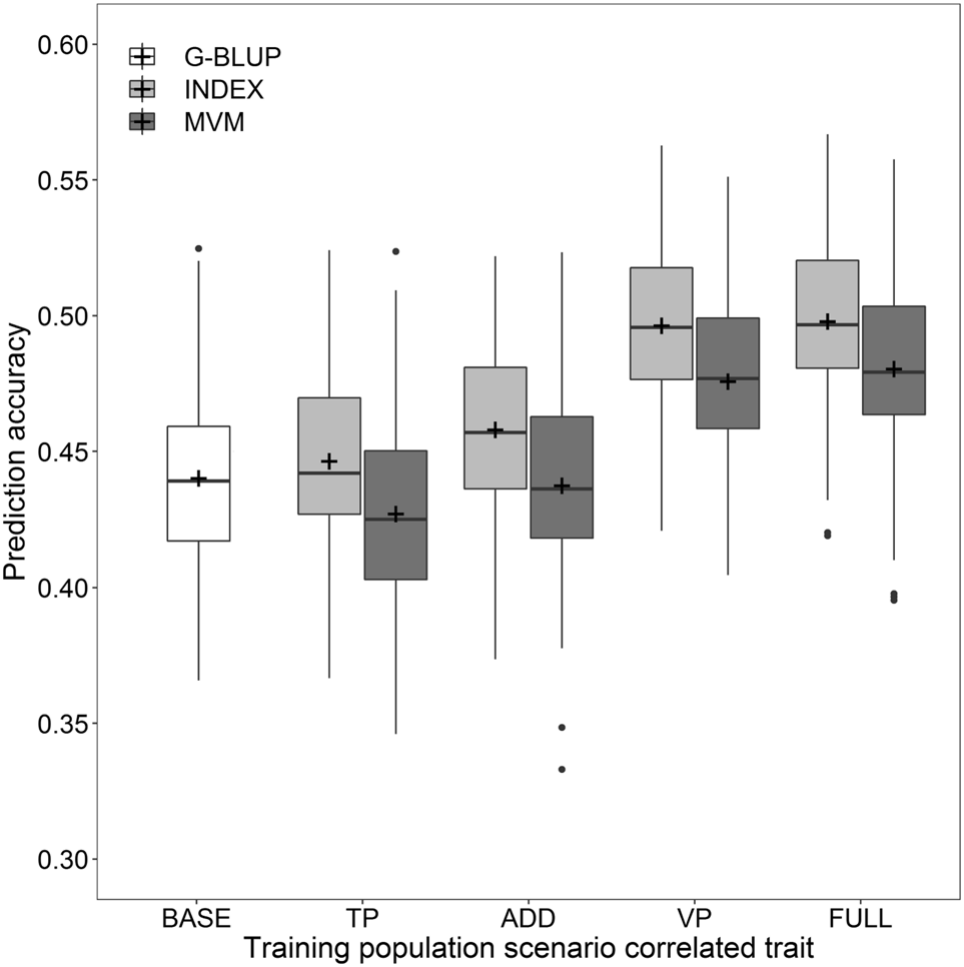
Prediction accuracy for multi-trait genomic selection fitting a multi-trait mixed model (MVM) or employing a model-based selection index approach (INDEX), and the comparison with the corresponding single-trait prediction (G-BLUP). The baseline accuracy without modelling correlations between traits (BASE) was compared with multi-trait prediction scenarios where additional phenotypic data on the protein content was only available for the training population (TP), for both the training and validation population (VP), the training population as well as a large number of additional lines (ADD), and for all involved lines (FULL)

Apart from wheat storage protein composition or protein quality, the protein content or quantity is an important measure of quality in wheat breeding programs, plant production as well as in milling and food-processing. An assessment of the protein content can be conducted with high precision and is readily applicable on a large number of samples by taking advantage of rapid test such as NIRS. Exploiting the relationship between the protein content and dough rheological parameters related to baking quality might consequently be an interesting strategy for improving the prediction accuracy of a genomic selection approach. However, cross-validation with multi-trait prediction models based merely on phenotypic information of training populations that were phenotyped for both protein content and dough rheology showed no benefit in comparison to single-trait prediction models (Fig. 4).

Notwithstanding, the prediction accuracy might be increased by including a large number of additional lines that have been genotyped and phenotyped for protein content in multi-environment trials though were not selected for further testing. This scenario is common to most wheat breeding programs and the retained phenotypic data of the additional lines could be efficiently salvaged by doubling training populations size of the protein content, i.e., the correlated trait in multi-trait prediction models, which resulted in, however, no increase of prediction accuracy (*r* = 0.44) compared with the single-trait prediction model (*r* = 0.44) trained merely with phenotypic data of the dough rheological traits (Fig. 4).

Another interesting scenario in plant breeding is the availability of an easy to score correlated trait to the actual traits of interest for the validation population. Possessing prior information of the protein content enabled a marked increase of the prediction accuracy of dough rheological traits under this condition (*r* = 0.48), while a combination of this and the previous scenario for increasing the training population size did not further effect the average prediction accuracy (*r* = 0.48). However, we observed higher prediction accuracies the multi-trait prediction model that melted the genomic estimated breeding values of the protein content and dough rheological traits by the model-based selection index method described in this study. The prediction accuracy of a model including a large number of additional lines phenotyped for protein content was elevated by this method (*r* = 0.46) as was it when prior information of the protein content was available (*r* = 0.50). The model-based selection index method was furthermore computationally far less demanding and did not lead to convergence problems that often occurred with the completely unstructured multivariate linear mixed models, hence we used this method for all further multi-trait predictions.

The forward prediction of 2014–2016 using this multi-trait prediction model showed a clear benefit over the standard RR-BLUP model of approximately 10.25% in average prediction accuracy when increasing the training population size for correlated trait protein content with additional thoroughly phenotyped lines. The increase in prediction accuracy was especially marked for dough development, water uptake, and farino quality number as well as extensibility and such a prediction strategy might be appropriate for an early generation genomic selection, where protein content data obtained from observation plots or preliminary yield trials might sometimes be very limited or of rather low quality depending on the trial. A similar improvement could be achieved by the W-BLUP, where the beneficial effect of upweighting the *Glu*-*1* loci markers was especially pronounced for resistance to extension and dough energy but we observed also some merit for dough stability and the farino quality number (Table 3). These results refer to the optimal W-BLUP models of this study, where merely *Glu*-*1* loci markers that explained more than 5.00% per cent of the genetic variance based on cross-validation in the basis population 2009–2013 were used as fixed effects. The *Glu*-*D1* locus with two alleles coding the subunits 5 + 10 and 2 + 12 played thereby a major role, followed by the *Glu*-*A1* locus that especially explained a substantial variation for dough stability. The *Glu*-*B1* locus on the other hand had the lowest importance most likely as mostly the 7 + 8 and 7 + 9 subunits occurred in the analysed material, which both have a very similar effect on protein quality. Combining the merits of a multi-trait prediction and modelling major QTL as fixed effects into a multi-trait W-BLUP model finally gave the highest average prediction accuracy (*r* = 0.47) surpassing the prediction accuracy of a standard RR-BLUP model by 20.50%, while the advantage in prediction accuracy varied between 7 and 61% for the individual traits (Table 3).

**Table 3:** Explained genetic variance estimated by cross-validation of the *Glu*-*1* loci in the basis population 2009–2013, and prediction accuracy for each dough rheological trait in the forward prediction of 2014–2016

Parameter	Explained genetic variance (%)			Model prediction accuracy			
	Glu-A1	Glu-B1	Glu-D1	RR-BLUP	W-BLUP^a^	INDEX^b^	MW-BLUP^c^
Water uptake	6.0	0.0	0.0	0.40	0.41	0.43	0.43
Development	12.6	1.4	1.9	0.36	0.36	0.40	0.39
Stability	30.4	4.0	33.9	0.38	0.45	0.41	0.46
Quality number	18.0	1.8	29.5	0.34	0.35	0.43	0.38
Resistance	2.4	0.6	7.3	0.30	0.49	0.31	0.49
Extensibility	1.5	0.0	0.3	0.40	0.40	0.46	0.46
Energy	6.3	0.0	12.7	0.53	0.61	0.56	0.63
Average	6.3	1.3	7.3	0.39	0.44	0.43	0.47

^a^Glu1 markers that explained more than 5.00% of the genetic variance were modelled as fixed effects

^b^Multi-trait model extending the training population with additional lines phenotyped for protein content

^c^Multi-trait W-BLUP model combining both prior prediction model extensions

**Fig. 5:**
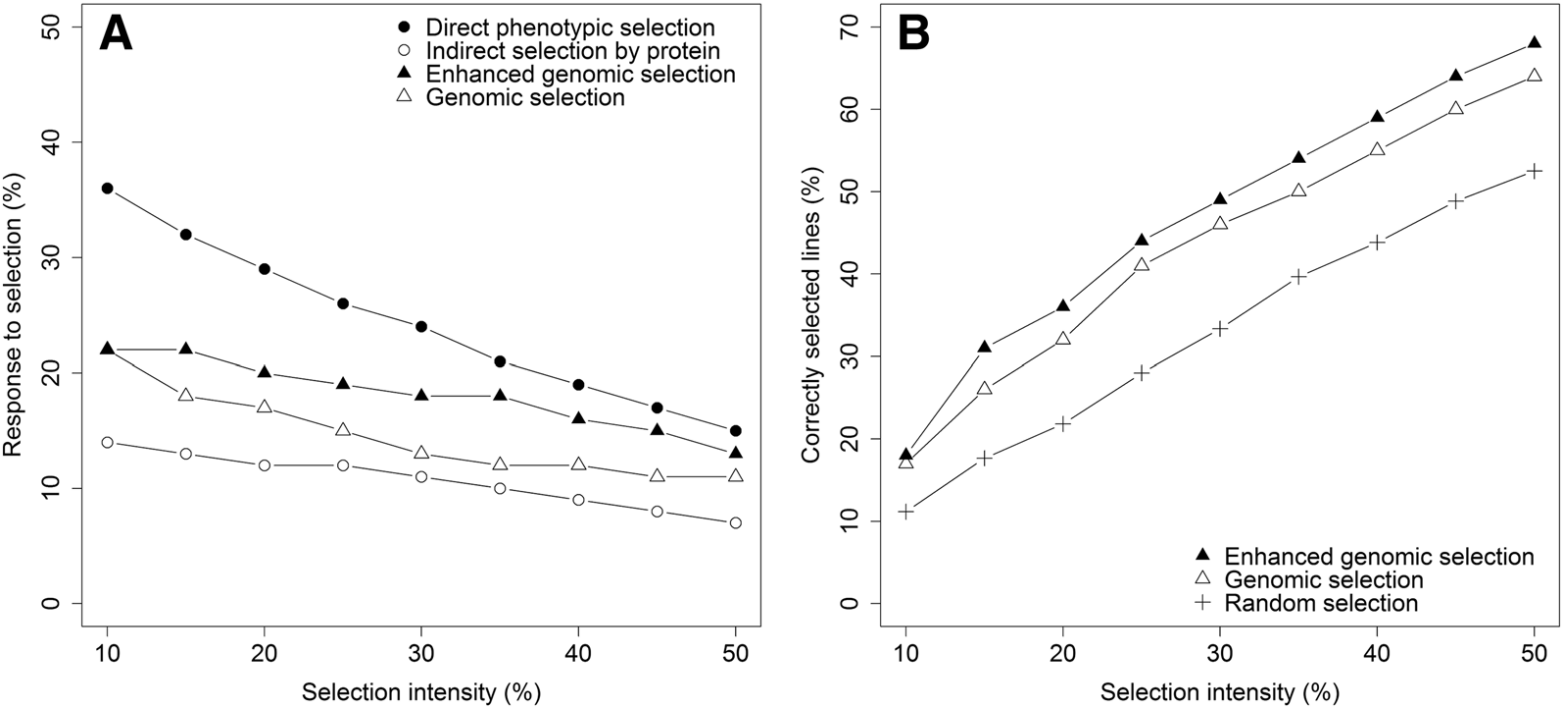
Response to selection averaged across all analyzed dough rheological traits using direct and indirect phenotypic selection as well as the comparison with genomic selection and the advantage of using the model-based selection index approach that additionally included markers associated with the *Glu*-*1* loci as fixed effects (**a**) as well as the number of correctly selected lines using the mentioned genomic selection approaches compared with a random selection (**b**)

**Fig. 6:**
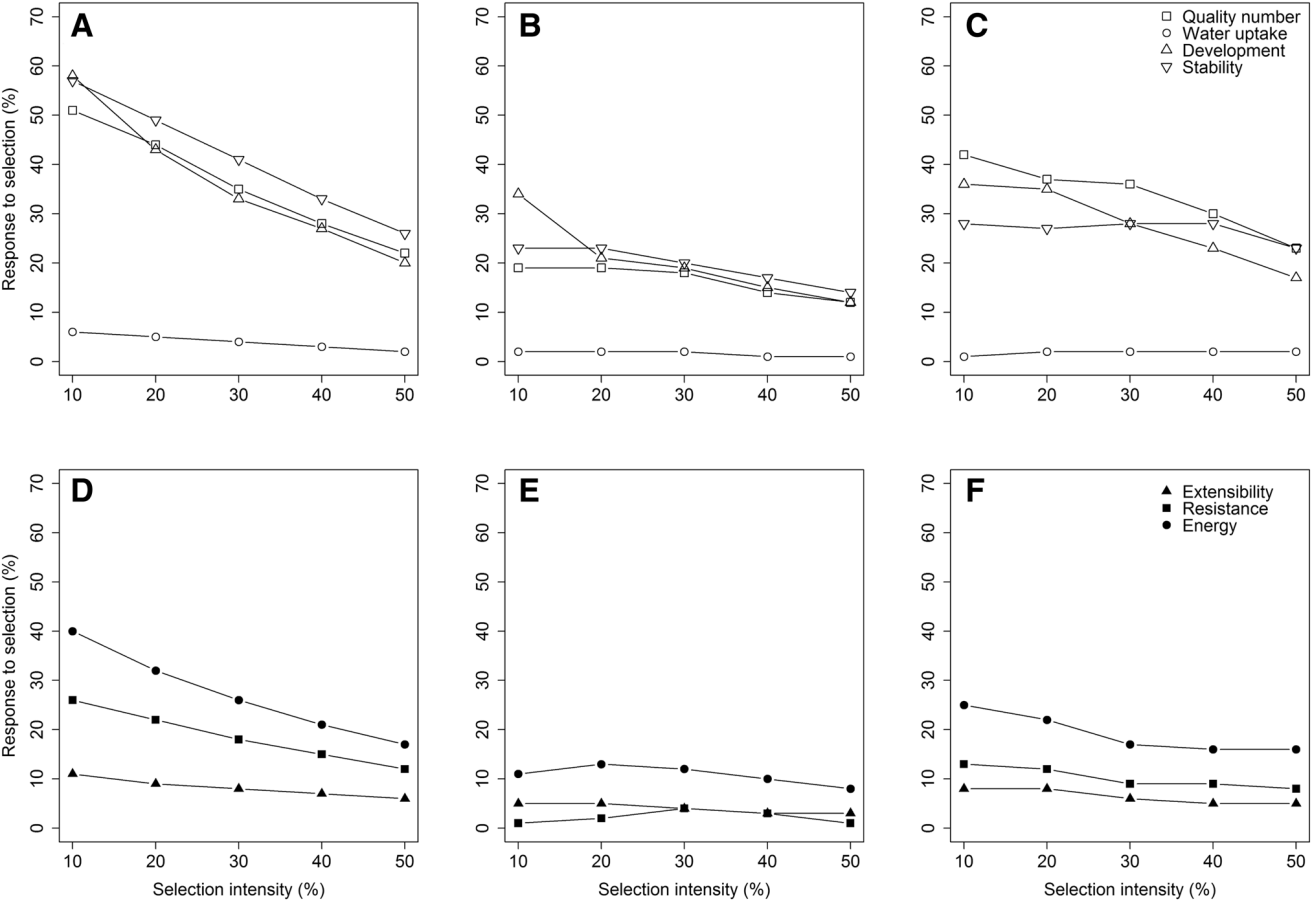
Response to selection for the individual rheological traits using direct (**a**, **d**) and indirect phenotypic selection (**b**, **e**) as well as the model-based selection index approach that additionally included markers associated with the *Glu*-*1* loci as fixed effects (**c**, **f**)

As expected the increase in prediction accuracy using the enhanced W-BLUP also led to a higher response to selection in terms of relative superiority in average trait performance of genomically selected subpopulations in comparison to the average trait performance of all lines in a given independent validation population (Fig. 5a). Although the results were quite promising for many dough rheological traits (Fig. 6), there was also great interest in comparing genomic selection with indirect phenotypic selection using an easy to phenotype correlated trait such protein content to pre-select germplasm for baking quality. We found a clear advantage of genomic selection in this comparison and a nearly twice as high response to selection in comparison to indirect selection by protein content for these baking quality related traits. However, a much larger response to selection was estimated for direct phenotypic selection of dough rheological traits even in this very unbalanced trial series due to the high heritability of these quality traits. Given these results, it is however of foremost importance to take the fact into consideration that dough rheology or baking quality can only be tested in very late stages of variety development in most wheat breeding programs. The applicable selection intensity for a direct phenotypic selection would accordingly be approximately around 50% of the retained lines, while in earlier generations where genomic selection is applied it would be possible to select much stricter, e.g., the 10% of lines with the highest performance. Although, the highest performing, i.e., most extreme lines could be equally well identified by both the baseline RR-BLUP and enhanced W-BLUP model (Fig. 5a), the latter showed a clear superiority over a large range of selection intensities and was more suited for identifying the correct lines in the respective selected fractions (Fig. 5b).

## Discussion

This study concentrated on genomic selection for labour-intensive and costly to assess dough rheological traits in order to support the improvement of baking quality in bread wheat breeding. The prospects of this approach were examined in various cross-validation schemes and a forward prediction with three independent validation populations using a large population of bread wheat lines tested across multiple years for dough rheological traits as well as their protein content. Apart from these prediction scenarios, several extensions of the genomic prediction models were investigated including multi-trait models and the exploitation of prior knowledge about the underlying trait genetic architecture.

### Integrating marker-assisted and genomic selection by knowing the trait genetic architecture

It is commonly accepted that the glutenins and gliadins strongly influence dough properties and baking quality due to the unique property of this cereal protein fraction of building a gluten network in a flour–water mixture (Shewry et al. 1995), which enables gas retention during dough formation allowing the dough to rise (Shewry et al. 2003; Weipert 2006; Rasheed et al. 2014). Apart from yield, disease resistance, and stress tolerance, the genetic improvement of quality traits is an important part of many national and international wheat breeding programs (Battenfield et al. 2016; Guzmán et al. 2016a; Liu et al. 2016; Würschum et al. 2016). Hence, great efforts were undertaken to decipher the genetic basis of storage protein quality of wheat, and it has been shown that amongst others the high molecular weight subunit composition of the glutenins strongly influences bread-making quality (Payne et al. 1987; Lukow et al. 1989; Rogers et al. 1989). The *Glu*-*1* loci found on the long arm of the homeologous chromosome pairs 1A, 1B and 1D associated with dough viscosity (Payne 1987) were found to be of especially large importance by many genetic studies for conferring dough resistance to extension (Kuchel et al. 2006; Mann et al. 2009; Tsilo et al. 2011; Cooper et al. 2016; Krystkowiak et al. 2016) and the respective favourable alleles also lead to a stronger gluten network thereby improving dough-mixing properties (Tsilo et al. 2013).

Accordingly, markers associated with the *Glu*-*1* loci have been used for predicting wheat quality traits by marker-assisted selection (Eagles et al. 2002; Kuchel et al. 2007; Oury et al. 2010) and also showed some merit for this purpose in our study. Analogous to alveograph W values (Oury et al. 2010) some rheological traits like the dough energy of the extensogram could in this way be predicted with a sufficient accuracy (*r* = 0.48), while other traits like dough extensibility and water uptake had a very low prediction accuracy or were not predictable at all, as the *Glu*-*1* loci did not explain a sufficient amount of genetic variance for the respective traits. A potential explanation could be that these traits are influenced by the large number of other genes associated with quality in wheat, amongst others the known *Gli*-*1* loci coding for gliadins associated with dough quality (Plessis et al. 2013; Sherman et al. 2014; Würschum et al. 2016) or genes that are directly influencing baking quality like *wbm* (Furtado et al. 2015; Guzmán et al. 2016b). Furthermore, a substantial number of small effect loci without candidate gene information have also been identified in numerous genetic mapping studies suggesting a complex inheritance of quality in wheat (Bordes et al. 2011; Reif et al. 2011; Tsilo et al. 2011, 2013; Cabrera et al. 2015), which indicates the worth of a genomic selection approach that takes both these small and medium effect loci as well as major QTL into account. Accordingly, we observed a substantial benefit of genomic over classical marker-assisted selection for dough rheological traits that is comparable to previous reports about genomic selection for milling and baking quality related traits (Battenfield et al. 2016; Liu et al. 2016).

Genomic selection is commonly used to predict complex quantitatively inherited traits with low and medium heritability in early generations of variety development when the available phenotypic information of important traits like grain yield is limited, and the usage of genomic estimated breeding values could substantially improve genetic gains by supporting breeders in their selection decisions (Auinger et al. 2016; Michel et al. 2016; Sallam and Smith 2016). Although, baking quality traits have usually a high heritability their assessment is often time-consuming, labour-intensive, costly, and too less plant material, i.e., grains are available from each selection candidate in early generations forcing breeders to postpone thoroughly quality testing into later generations of variety development. Genomic selection has on the other hand the great advantage to enable a pre-selection of high performing lines in a much broader population 2–3 years before conducting these costly tests, thereby promoting the selection of lines that combine desirable quality characteristics and grain yield. Integrating major genes as fixed effects into genomic prediction models has furthermore been shown to improve such a genomic selection approach for plant morphological and disease resistance traits (Bernardo 2014; Zhao et al. 2014; Arruda et al. 2016), and we could verify this W-BLUP method with the *Glu*-*1* loci markers that were associated with dough rheological traits in our study. Fine-tuning these W-BLUP models includes an appropriate compromise between marker number and proportion of explained genetic variance, which most likely depends on the breeding material and can to some extent be guided by an appropriate cross-validation in the training population. A major prerequisite is of course that alleles of these QTL have not been fixed in the respective breeding population yet. Extending this idea, Spindel et al. (2016) suggested to integrate de novo mapped marker-trait associations into genomic prediction models. Liu et al. (2016) could though not find any advantage of this method in the analysis of a large hybrid wheat population for quality traits, while other studies reported a significant increase in prediction accuracy of this method (Boeven et al. 2016; Moore et al. 2017). The increase in prediction accuracy using the same population for marker-trait associations discovery and subsequent prediction model validation has been termed the inside trading effect by Arruda et al. (2016), and is the result of selecting predictors before leaving observations that are supposed to be unobserved out, leading consequently to an overfit of the respective prediction models to the training data. Accordingly, the prediction accuracy was even negatively affected when such W-BLUP models that included de novo found markers were used for predicting the three independent validation populations, which firstly showed though great promise in the cross-validation within the estimation set. However, we also did not find an advantage of de novo found markers in the independent validation, which seemed to be promising at first based on the correctly applied cross-validation using a validation set of left-out lines. Possible reasons that these latter de novo found markers could not be validated might be a too small population size for mapping combined with too low marker coverage for accurately mapping the underlying loci, linkage phase changes between training and validation population or the false positive rate. Nevertheless, these issues suggest a prudent interpretation of interesting markers identified in GWAS, and marker-trait associations should be validated with data that was left-out for mapping. We suggest thus favouring known major QTL like *Fr*-*2* for frost tolerance (Erath et al. 2017; Würschum et al. 2017) or *TaPHS1* for pre-harvest sprouting (Moore et al. 2017) when predicting complex traits with W-BLUP models in bread wheat and other species. Important genes associated with dough rheological parameters like the *Glu*-*1* loci could nevertheless be readily identified by GWAS (Zheng et al. 2009); however, it often fails to detect rare variants like the *wbm* gene (Furtado et al. 2015; Bernardo 2016; Guzmán et al. 2016b). Mapping within bi-parental populations using the same marker system as employed in the respective breeding program might thus be a more appropriate strategy for finding new interesting marker-trait associations, which could subsequently be integrated into the genomic selection framework taking advantage of the vast results and experiences gained in QTL mapping during the last two decades.

### Enhancing genomic selection by utilizing the association between protein quality and quantity

Apart from the wheat storage protein composition dough rheological and thus baking quality is also determined by protein quantity. Accordingly, we observed strong genetic and phenotypic correlations between protein content with different traits especially dough water uptake, development and extensibility. Whereas the glutenin loci also play a role in the expression of the latter mentioned traits (Zheng et al. 2009; Tsilo et al. 2013), they are to a larger extent controlled by loci associated with the gliadin protein fraction and the gliadin/glutenin ratio (Bekes 2012a, b; Plessis et al. 2013; Sherman et al. 2014) probably causing this strong correlation with the protein content (Osman et al. 2012; Marti et al. 2015).

Some response to indirect selection based on protein content can thus be expected for these quality traits where a marker-assisted selection with *Glu*-*1* loci markers was not applicable, especially for dough extensibility which is highly dependent on the gliadin protein fraction. However, for optimal selection breeders should also consider the complex interaction of protein fractions, which was reflected by the negative relationship between extensibility and resistance to extension found in our and previous studies (Zanetti et al. 2001; Bordes et al. 2008). The resistance to extension generally increases if the glutenin fraction prevails, i.e., with a lower gliadin/glutenin ratio (Melnyk et al. 2012) and baking quality traits like loaf volume are thus dependent on both protein fractions. The same consideration is valid for traits related to dough processing, where genotypes with a prevailing gliadin fraction show a fast water uptake and dough development but an often insufficient dough stability (Weipert 2006). Given these interactions, a simultaneous improvement of extensibility and resistance to extension could be achieved by selecting for dough energy or area under the extensogram curve as an integrated measure that takes both resistances to extension and extensibility into account. Likewise, mixing properties and thus the stability of gluten networks could be improved by utilizing the farino quality number as an integrated index. These two traits showed also a high prediction accuracy using a genomic selection approach, which was superior to indirect selection by the protein content. Although the information of such single point values might be limited (Dobraszczyk and Morgenstern 2003), they could be used to pre-select lines with desirable trait combinations before complete dough rheological profiles can be created for a final selection decision of lines entering variety registration trials.

Notwithstanding, the protein content is an easy to measure rapid test that can be applied to a large number of samples in a short time period. Vast phenotypic information of a large number of lines can thus be expected in wheat breeding programs, which could be used for enlarging the training population size in a genomic selection approach. This is certainly beneficial for wheat breeders as a larger training population size has been shown to increase the prediction accuracy in numerous genomic selection studies (Heffner et al. 2011; Battenfield et al. 2016; Nielsen et al. 2016), and exploiting the strong correlation between dough rheological traits and the highly predictable protein content in a multi-trait prediction model gave accordingly some increase in prediction accuracy if the training population size of the correlated trait protein content exceeded the one of the main trait of interest. On the other hand, multi-trait prediction models performed often very similar to single-trait predictions or were even slightly inferior when the training population was comprised only of lines that were phenotyped in parallel for both traits (Jia and Jannink 2012; Guo et al. 2014; dos Santos et al. 2016). Hence, we suggest that information from the vast number of additional lines phenotyped for correlated traits should be included into multi-trait prediction models to fully exploit the merit of such models. This issue is especially interesting in wheat breeding programs, where breeders can strongly profit from the protein content as a highly heritable as well as easy to phenotype trait which is genetically highly correlated with traits related to baking quality. This advantage could be further extended when the validation or selection population was already phenotyped for the correlated trait (Jia and Jannink 2012; Hayes et al. 2017). This scenario gave a strong increase in prediction accuracy in our study, and could effectively be used to enhance selection in advanced generations when reliable protein content data is already available, though too less plant material for dough rheological test. Additionally, the according genomic predictions could support the choice in combination with the sedimentation value as an intermediate quality analysis step which material should actually be sent to the laboratory for in depth quality analysis. This is another important decision in quality breeding, and we suggest analysing a broad sample from all quality classes in order to avoid a bias and thereby keeping a high prediction accuracy for a successful long-term selection strategy (Zhao et al. 2012).

## Conclusions

This study focused on the merit of genomic selection for the genetic improvement of laborious to phenotype dough rheological traits that are related to baking quality in bread wheat. Genomic selection showed a superior performance over marker-assisted and indirect phenotypic selection and could be enhanced by exploiting prior knowledge about the underlying trait genetic architecture for the estimation of genomic breeding values. It was also shown that great care must be taken when upweighting the effect of certain markers in the prediction models and a trait-specific fine-tuning by the proportion of explained genetic variance is advisable. Additional fine-tuning of the predictions was furthermore possible by employing multi-trait prediction models when increasing the training population size of the rapid to phenotype protein content that served in this case as highly heritable and correlated trait. Finally, a genomic selection approach revealed a major benefit over classical selection methods for many quality traits as it would allow a 2–3 years earlier selection for the often costly, labour-intensive and time-consuming assessment of line performance by sophisticated quality tests. This considerable advantage, combined with a higher applicable selection intensity in early generations could support breeders in developing new bread wheat varieties that efficiently combine superior baking quality with comparatively higher grain yield.

### **Author contribution statement**

SM wrote the manuscript and analysed the data. CA and JH supported in the statistical analysis. MG performed the dough rheological analysis and CK supported in the dough rheological interpretation of the results. FL, DE, and BA designed the field trials and collected the phenotypic data in the field. FL and HB initiated and guided through the study. All authors read and approved the final manuscript.

## Electronic supplementary material

Below is the link to the electronic supplementary material.

Supplementary material 1 (PDF 228 kb)

Supplementary material 2 (CSV 5908 kb)

Supplementary material 3 (R 10 kb)

Supplementary material 4 (PDF 238 kb)
