# Supplementary material for: Improving the baking quality of bread wheat by genomic selection in early generations
Source: Theor Appl Genet. 2017 Oct 23;131(2):477–93. doi: 10.1007/s00122-017-2998-x (PMC5787228; doi:10.1007/s00122-017-2998-x)
Supplement: Supplementary file 1 — Supplementary material 1 (PDF 228 kb) [file 122_2017_2998_MOESM1_ESM.pdf]

Online Resource 1

Article Title: Improving the baking quality of bread wheat by genomic selection in early generations

Journal: Theoretical and Applied Genetics

Authors: Sebastian Michel, Christian Kummer, Martin Gallee, Jakob Hellinger, Christian Ametz, Batuhan Akgöl, Doru Epure, Franziska Löschenberger, Hermann Buerstmayr

Name, affiliation, and email of corresponding author:

Sebastian Michel
Department for Agrobiotechnology (IFA-Tulln)
Institute for Biotechnology in Plant Production
University of Natural Resources and Life Sciences, Vienna (BOKU)
Konrad-Lorenz-Str. 20, 3430 Tulln, Austria
e-mail: sebastian.michel@boku.ac.at

Table S1 Overview of the subdivision of the dough rheological analysed population of lines into five sets as well as the additional set of lines phenotyped for protein content in multi-environment trials.

		Phenotyped traits		
Set	Years	Protein content	Rheological	Lines[†]
$Total_{Rheo}$	2009-2016	x	x	401
$Basis_{Rheo}$	2009-2013	x	x	191
$Independent_{Rheo}$	2014	x	x	70 (70)
	2015	x	x	43 (79)
	2016	x	x	97 (125)
$Addition_{Protein}$	2009-2013	x		439
Sum				840

[†]Number of unique lines in each set and the number of lines used for the analysis in parenthesis.
