# Supplementary material for: Improving the baking quality of bread wheat by genomic selection in early generations
Source: Theor Appl Genet. 2017 Oct 23;131(2):477–93. doi: 10.1007/s00122-017-2998-x (PMC5787228; doi:10.1007/s00122-017-2998-x)
Supplement: Supplementary file 4 — Supplementary material 4 (PDF 238 kb) [file 122_2017_2998_MOESM4_ESM.pdf]

Online Resource 4

Article Title: Improving the baking quality of bread wheat by genomic selection in early generations

Journal: Theoretical and Applied Genetics

Authors: Sebastian Michel, Christian Kummer, Martin Gallee, Jakob Hellinger, Christian Ametz, Batuhan Akgöl, Doru Epure, Franziska Löschenberger, Hermann Buerstmayr

Name, affiliation, and email of corresponding author:

Sebastian Michel
Department for Agrobiotechnology (IFA-Tulln)
Institute for Biotechnology in Plant Production
University of Natural Resources and Life Sciences, Vienna (BOKU)
Konrad-Lorenz-Str. 20, 3430 Tulln, Austria
e-mail: sebastian.michel@boku.ac.at

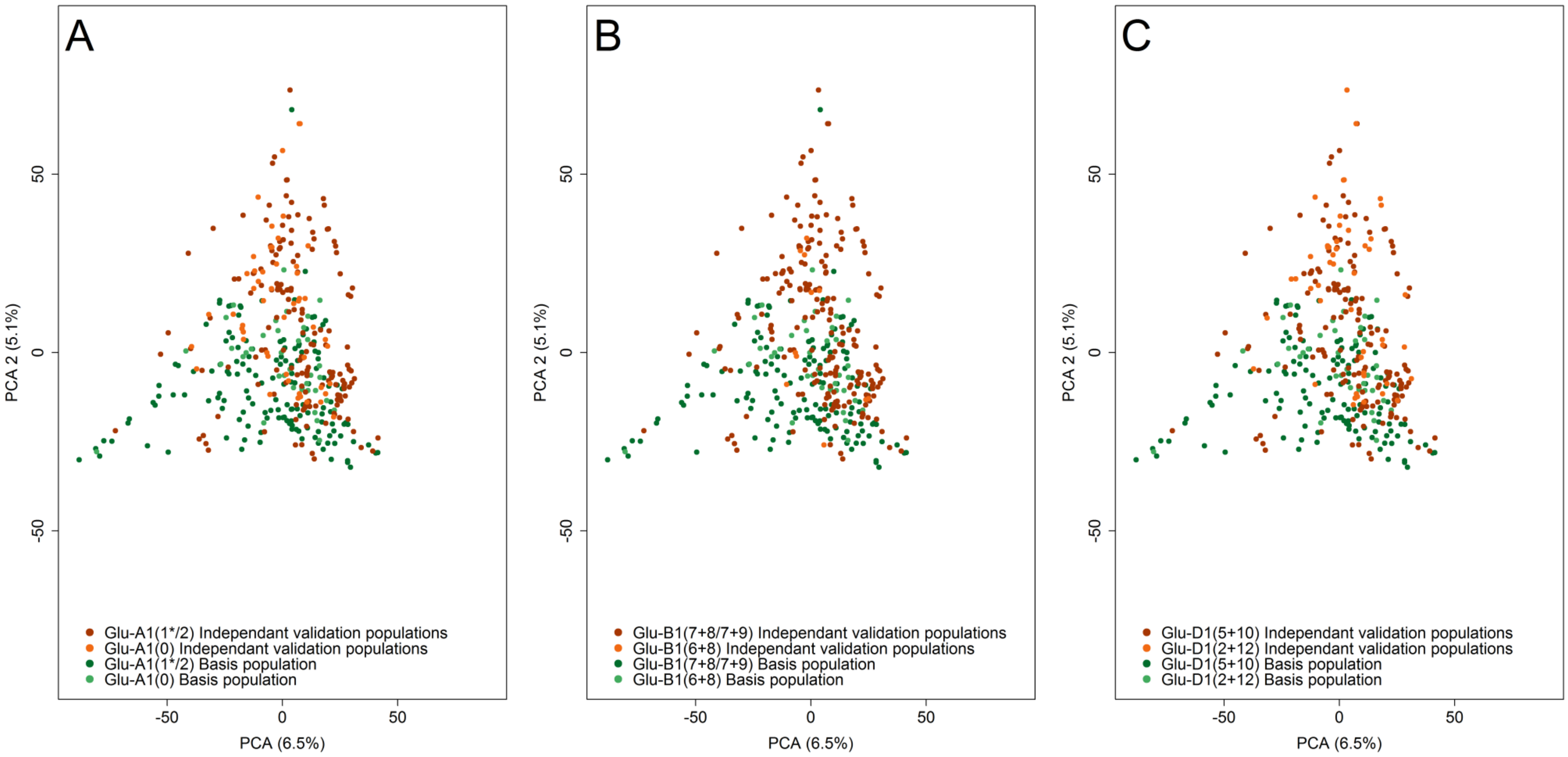

Fig. S1 Population structure for the basis population 2009-2013 and independent three independent validation populations 2014-2016 with respect to their alleles at the *Glu-A1* (A), *Glu-B1* (B), and *Glu-D1* (C) marker locus.
